# Antiproliferative Activity of the Isofuranonaphthoquinone Isolated from *Bulbine frutescens* against Jurkat T Cells

**DOI:** 10.1155/2014/752941

**Published:** 2014-01-16

**Authors:** Penelope Tambama, Berhanu Abegaz, Stanley Mukanganyama

**Affiliations:** ^1^School of Pharmacy, College of Health Sciences, University of Zimbabwe, Mount Pleasant, Harare, Zimbabwe; ^2^The African Academy of Sciences, P.O. Box 24916, Nairobi, Kenya; ^3^Biomolecular Interactions Analyses Group, Department of Biochemistry, University of Zimbabwe, P.O. Box MP 167, Mount Pleasant, Harare, Zimbabwe

## Abstract

Cancer is a major public health burden in both developed and developing countries. The quinone moiety has been shown to possess antitumor activity and several cancer drugs in clinical use contain this entity. The effect of isofuranonaphthoquinone isolated from *Bulbine frutescens* on Jurkat T cells was determined. Cells were exposed to the isofuranonaphthoquinone (IFNQ) at different concentrations. Significant antiproliferative effects were observed which were comparable to that of the anticancer drug 1,3-bis(2-chloroethyl)-1-nitrosourea (BCNU). A combination of IFNQ with BCNU produced synergistic effects which were observed after 72 hrs. It was also observed that combining IFNQ with reduced glutathione abolished the anticancer activity of the compound. It is, therefore, proposed that the isofuranonaphthoquinone may exert part of its effect by producing reactive oxygen species resulting in death of the cells as the effects of this compound were antagonized by reduced glutathione. An investigation on the effects of isofuranonaphthoquinone on glutathione transferase (GST) activity and drug efflux pumps showed that this compound exhibited inhibitory effects on both the GST and the drug efflux pumping activities. Thus, the isofuranonaphthoquinone showed cytotoxicity, works through inhibition of some cellular mechanisms, and could present a potential source of lead compounds for anticancer drug development.

## 1. Introduction

Cancer is a term describing conditions characterized by uncontrolled cellular proliferation and differentiation [1]. There are over 100 different types of cancer, and each is classified by the type of cell that is initially affected. Tumors can grow and interfere with the digestive, nervous, and circulatory systems and they can release hormones that alter body function [2]. In the case of leukemia, the cells prohibit normal blood function by abnormal cell division in the blood stream [3].

Cancer is the second leading cause of death, after heart disease, where one in four deaths is due to cancer [5, 6]. The global burden of cancer is large and growing larger each passing year. Each year, more than 11 million people are diagnosed with cancer worldwide. It is estimated that by the year 2020, this number will increase to 16 million [7]. In addition, cancer causes more than 8 million deaths each year or approximately 13 percent of all deaths worldwide. In most developed countries, cancer accounts for more than 20 percent of all deaths. In less developed countries, overall cancer rates are generally lower and cancer accounts for a lower percentage of deaths. In Zimbabwe, the Zimbabwe Cancer Registry (ZCR) is a multiple source cancer registration and surveillance system that is population-based for Harare, the capital city [8]. The registry also accepts notifications that originate from outside its target population of Harare city. The additional information is used to estimate the pattern and burden of cancer in the country. According to ZCR, the total number of new cases of cancer recorded among Zimbabweans in 2006 was 4175 comprising 1842 (44.1%) males and 2333 (55.9%) females. The leading cause of cancer among Zimbabwean black men in 2006 was Kaposi sarcoma (KS) (23.8%). This was followed by prostate (13.1%), eye (6.5%), non-Hodgkin lymphoma (NHL) (6.2%), esophagus (6.2%), liver (4.9%), stomach (3.8%), lung (3.0%), bladder (2.9%), and nonmelanoma of skin cancer (2.7%).

Cancer develops when the damaged cell's ability to commit suicide is impaired or when the cell loses responsiveness to normal growth control. The damaged cell begins to multiply and gives rise to a clone of mutated daughter cells. Tumors that stay in one spot and demonstrate limited growth are generally considered to be benign [9]. Exposure to carcinogenic chemicals can increase one's risk of developing cancer. The polycyclic aromatic hydrocarbons contained in tar of cigarette smoke are carcinogenic [10]. Smoking is strongly associated with the development of carcinomas of the lungs (squamous and small cell variants). In the industry, exposure to products of rubber and aniline dye can increase the risk of someone developing cancer. Chemicals such as azo dyes and *β*-naphthylamine cause cancers of the kidney, pelvis, ureter, and bladder because they are excreted in urine [9]. Aflatoxin, a product of the fungus *Aspergillus flavus*, contaminates poorly stored cereals and nuts [11]. It is associated with hepatocellular carcinoma.

It is very unfortunate that alkylating chemotherapy agents have long-term effects on the DNA, changing its configuration and leading to difficulty when a cell undergoes mitosis or meiosis. Alkylating agents intercalate into DNA causing mutations due to mispairing of nucleotides [10]. Thus, alkylating agents are toxic, mutagenic, carcinogenic, and teratogenic [12] as they tend to react with bases in proteins and inactivate enzymes. Drug resistance associated with chemotherapy is a major drawback in achieving therapeutic outcomes [13]. There are different mechanisms by which resistance emerges and these include efflux pumps on the cell membranes, decreased drug uptake due to P-glycoprotein action, altered drug targets, and repair of damaged DNA [14].

Reactive oxygen species have been implicated in the etiology of many diseases including cancer [15]. Free radicals cause tissue damage via oxidative stress and the overproduction of these agents causes inflammation, alters DNA and causes the oxidation of lipids and proteins [16]. Altered DNA can lead to mis-pairing of the nucleotides and results in mutations. Mutations can lead to cancer development. Cancer is a result of mutations that affect the tumor suppressor genes such as p53 or the proto-oncogenes whose products are involved in cell signaling. The mechanism that follows depends on factors such as the type of active oxygen species involved and the intensity of stress [17].

The main cellular targets affected by oxidative stress are DNA, phospholipids, proteins, and carbohydrates on the cell membrane [18]. When DNA is oxidized and injured, it will have the capacity to induce genetic mutation. It is now apparent that some telomere genes are highly susceptible to mutation in the presence of free radicals [16]. Tumor suppressor genes such as p53 and cell cycle-related genes may also suffer DNA damage [19]. Normal aerobic metabolism gives rise to oxidants which are active and potentially dangerous even under normal physiological conditions [20]. Inflammation, exposure to foreign compounds, and radiation can also give rise to oxidants [21]. In relation to cancer development, interactions of reactive oxygen species generated with DNA are very important [22].

**Figure 1:**
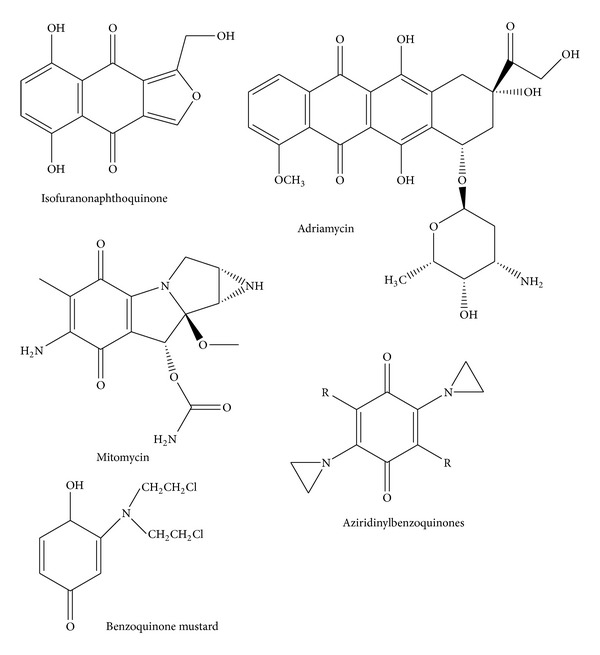
Structure of the isofuranonaphthoquinone isolated from *Bulbine frutescens* and other examples of quinone-containing antitumor agents (adapted from 67).

Natural products are widely used in cancer chemotherapy. The search for anticancer agents from plant sources started in the 1950s with the discovery and development of the vinca alkaloids, vinblastine and vincristine, and the isolation of the cytotoxic podophyllotoxins [23]. Quinones are an important group of chemicals in the treatment of cancer. Quinone-containing compounds have been widely used for their antitumor and anticancer activity (Figure 1). However, problems such as toxicity and drug resistance have stimulated an intense demand and research efforts for the discovery of new and novel antitumor agents. In light of this, significant progress has been made in the screening of the quinone moiety for antitumor activity [24]. The redox cycling of the quinone moiety is implicated in the antitumor activity of anticancer compounds containing a quinone moiety [25].

Most of the currently used antineoplastic agents elicit their effects by directly damaging cellular DNA or through mechanisms involving the inhibition of DNA synthesis, the mitotic apparatus, or topoisomerases. DNA damage results from intercalation of antineoplastic agents into DNA. The semiquinone can generate reactive oxygen species close to DNA thereby causing damage [26]. The isofuranonaphthoquinone (Figure 1) occurs naturally as a phytochemical constituent of various plant species. It occurs in Bulbine plant species such as *Bulbine abyssinica*, *Bulbine capitata*, and *Bulbine frutescens *[27]. The genus *Bulbine* (Asphodelaceae) consists of 80 species found in Australia and Africa. These are succulent, caulescent, branched, rhizomatous, and caespitose or solitary geophytes. Biaryl anthraquinones, knipholone and isoknipholone, have been isolated from the roots of *Bulbine frutescens *as well as from other *Bulbine *species [28]. Knipholone, together with two new phenylanthraquinones (Gaboroquinones A and B), and a demethylknipholone glucoside have been isolated from the roots of plants growing in Botswana [29].

Plants products maintain the health and vitality of individuals and also cure diseases, including cancer without causing toxicity [30]. Natural compounds have substantial structural diversity and present new mechanisms of biological activity [31]. They provide more than 50% of the drugs available [32]. Molecules derived from nature play a dominant role in the discovery of leads for the development of conventional drugs used in human medicine. Previously we showed that the isofuranonaphthoquinone inhibited human recombinant glutathione transferase *in vitro*, an enzyme associated with anticancer drug resistance [33]. Since the quinone moiety on a drug has been shown to impart anticancer activity [24], we were interested in exploring further the biological activity of this compound on cancer cells. This anticancer activity is postulated to be mediated via a redox cycling pathway in which reactive oxygen species (ROS) produced would disrupt cell activities resulting in cell death. In this regard, the effects of combining isofuranonaphthoquinone with reduced glutathione on Jurkat T cells, as well as the effects of combining the compound with 1,3-bis-(2-chloroethyl)-1-nitrosourea (BCNU), were determined. We were also interested in assessing the mode of action of this compound in these cells by determining its effects on drug transport across the cell membrane.

## 2. Materials and Methods

### 2.1. Reagents and Chemicals

The substrates 1-chloro-2,4 dinitrobenzene (CDNB), monochlorobimane (MCB), and other chemicals and reagents were obtained from Sigma Chemical Company and Aldrich Chemical Company (St. Louis, MO, USA). The natural product compound, isofuranonaphthoquinone extracted from *Bulbine frutescens*, was obtained from Professor Berhanu Abegaz (University of Botswana, Botswana). This compound was extracted as described before [33].

### 2.2. Culturing of Jurkat T Cells

The Jurkat human T-cell lymphoma cell line was obtained from Sigma-Aldrich (Germany). The cells were maintained in a fully humidified Shelab incubator (Sheldon Manufacturing, Cornelius, OR, USA) containing 5% CO_2_, at 37°C at a density of approximately 5 × 10^5^ cells per mL in RPMI-1640 medium supplemented with 10% (v/v) FBS, 5 mg/mL streptomycin, 5000 IU/mL penicillin, 10 mg/mL neomycin, and 2.0 mM L-glutamine.

### 2.3. Cell Viability Assay

The Trypan blue dye exclusion assay was used in all the cell viability determinations carried out. Cells were incubated in duplicate per treatment in 12-well plates. For cell counting, each sample from the 12-well plates was counted in triplicate by taking 200 *μ*L of of cells and adding 100 *μ*L Trypan blue 4% in 96-well plates. A cell count would then be conducted under the microscope using a hemocytometer whereby dead and live cell numbers were recorded. Percentage cell viability was calculated using the following formula:

(1) $$\begin{array}{c} \%\text{Cell}\;\text{viability}=\frac{\text{Number}\;\text{of}\;\text{live}\;\text{cells}}{\text{Total}\;\text{number}\;\text{of}\;\text{cells}}\times 100\%. \end{array}$$

### 2.4. Determination of the Effects of IFNQ on Jurkat T Cells

Cell count was performed using the Trypan blue dye exclusion assay to determine cell viability in a volume of cells that contained 1 × 10^5^ cells/mL. The effects of the isofuranonaphthoquinone were determined in duplicate in a 12-well plate. The total volume for each well was 3 mL. Initially the cells were exposed to a highest concentration of 50 *μ*g/mL isofuranonaphthoquinone and this was shown to kill all cells. Concentrations of 0, 12.5, 16.7, 25, and 38 *μ*g/mL were then used in the later studies. The cells were incubated at 37°C in a humidified atmosphere with 5% carbon dioxide. Cell counts were done every 24, 48, and 72 hours using the Trypan blue dye exclusion assay. Cell viability was determined as described before.

### 2.5. Determination of the Effects of Combining IFNQ with Reduced Glutathione

Glutathione is an endogenous antioxidant and as such would possibly antagonise the pro-oxidant role of the test compound. In order to determine a possible mechanism of action of the compound, the effects exposing cells to this compound and reduced glutathione (GSH) were determined. Cells were exposed to media alone (negative control containing an equal amount of drug vehicle) IFNQ alone (25 *μ*g/mL), GSH alone (25 *μ*g/mL), BCNU 50 *μ*g/mL + GSH 50 *μ*g/mL, and IFNQ 25 *μ*g/mL + GSH 25 *μ*g/mL. The concentration of 25 *μ*g/mL IFNQ was based on a predetermined value that gave at least 50% viability of Jurkat cells. The total volume per well was made up to 3 mL with RPMI media and contained 1 × 10^5^ cells/mL. The plates were incubated at 37°C in humidified atmosphere at 5% carbon dioxide (Shelab incubator, Sheldon Manufacturing, Cornelius, OR, USA). Cell counts were conducted every 24, 48, and 72 hrs using the Trypan blue dye exclusion assay, and cell viability was determined. The combination index (CI) was calculated from the formula:

(2) $$\begin{array}{c} \text{CI}=\frac{\text{Inhibition}\;\text{of}\;\text{isofuranonaphthoquinone}+\text{BCNU}}{\text{Inhibition}\;\text{of}\;\text{BCNU}}. \end{array}$$

The CI values were then interpreted as follows: <0.5: synergism, >0.5 to 1.0: no interaction, and 1.0 to 4.0: antagonism [4].

### 2.6. Determination of the Reversibility of the Effects of Isofuranonaphthoquinone

To determine whether the effects of isofuranonaphthoquinone on Jurkat T cells are reversible or not, the cells were incubated with the IFNQ for 72 hrs. Cells at 1 × 10^5^ cells/mL were incubated with 0, 12.5, and 25 *μ*g/mL of IFNQ on a 6-well plate. After incubating for 72 hrs, a cell count was done. Cells were then centrifuged in 15 mL centrifuge tubes at a speed of 1000 rpm for 5 minutes (Rotofix 32, Hettich Zentrifugen, Tuttlingen, Germany). Cells were washed twice with the medium. The cells were resuspended in 6 mL fresh medium and incubated for another 72 hrs. After 72 hrs, another cell count was done to determine if the effects of IFNQ could be reversed.

### 2.7. Effects of IFNQ on Glutathione Transferases

Glutathione transferases are usually overexpressed in cancer cells [34] and have been linked to drug resistance. Cells pretreated with 0, 12.5, or 25 *μ*g/mL were used in the GST activity determination. Cells were resuspended with 1 mL of PBS (pH 7.2). The cells were then sonicated to break open the cell membranes so that GST activity could be determined using 96-well plates. The assay with 1-chloro-2,4-dinitrobenzene (CDNB) was adapted for measurement of absorbance with a SpectraMax 340 microplate spectrophotometer equipped with a kinetics mode (Molecular Devices, Sunnyvale, California, USA).

### 2.8. Determination of the Effect of Isofuranonaphthoquinone on Drug Efflux Pumps

MCB conjugates to glutathione inside the cell in a reaction catalyzed by cytosolic GSTs. The MCB-glutathione conjugate fluoresces at excitation wavelength 390 nm and emission wavelength of 478 nm [35]. The MCB-glutathione conjugate is then effluxed by the multidrug resistance associated protein (MRP1) pumps out of the cytosol. The accumulation of the conjugate in the cell is measured as the amount of fluorescence when the cells are lysed. A cell count of 2 × 10^6^ cells/mL were washed in Hank's buffered salt solution (HBSS) and centrifuged at 2000 rpm for 5 minutes. The resulting pellet was resuspended in ice-cold HBSS supplemented with 10 mM HEPES pH 7.4 and centrifuged at 2000 rpm for 5 minutes. The cells were loaded with 5 *μ*M monochlorobimane (MCB) in HBSS for 1 hr and incubated at 10°C in a refrigerated water bath. After incubation, the cells were washed twice with ice-cold HBSS pH 7.4 supplemented with 11.1 mM glucose. The cells were resuspended in HBSS supplemented with glucose and divided into six equal portions. Three tubes were incubated at 37°C, whilst the other three were incubated at 10°C. The tubes were treated with either 1 mM isofuranonaphthoquinone (IFNQ) or 1 mM 1-chloro-2,4-dinitrobenzene (CDNB). Samples were incubated for 1 hr at 37°C and 10°C and then centrifuged at 1000 rpm for 5 minutes. The supernatant (supernatant 1) was collected. Cells were resuspended in HBSS and sonicated to break open the cell membrane. The cells were centrifuged again and supernatant (supernatant 2) was collected. Fluorescence of the supernatants was measured at excitation and emission wavelengths of 390 nm and 478 nm, respectively, using a Shimadzu UV-1501 spectrophotofluorometer (Shimadzu Corporation, Kyoto, Japan).

### 2.9. Statistical Analysis

One-way analysis of variance test (ANOVA) with Dunnett's multiple comparison posttest was used to analyse the results. All columns of treatments were compared to the control. The values with a *P* value < 0.05 or less were considered statistically significant. Graphical and Statistical analyses were carried out using Graphpad Prism 5 Software (Version 5.0, Graphpad Software Inc., San Diego, USA).

## 3. Results

### 3.1. Effects of IFNQ on Cell Proliferation

**Figure 2:**
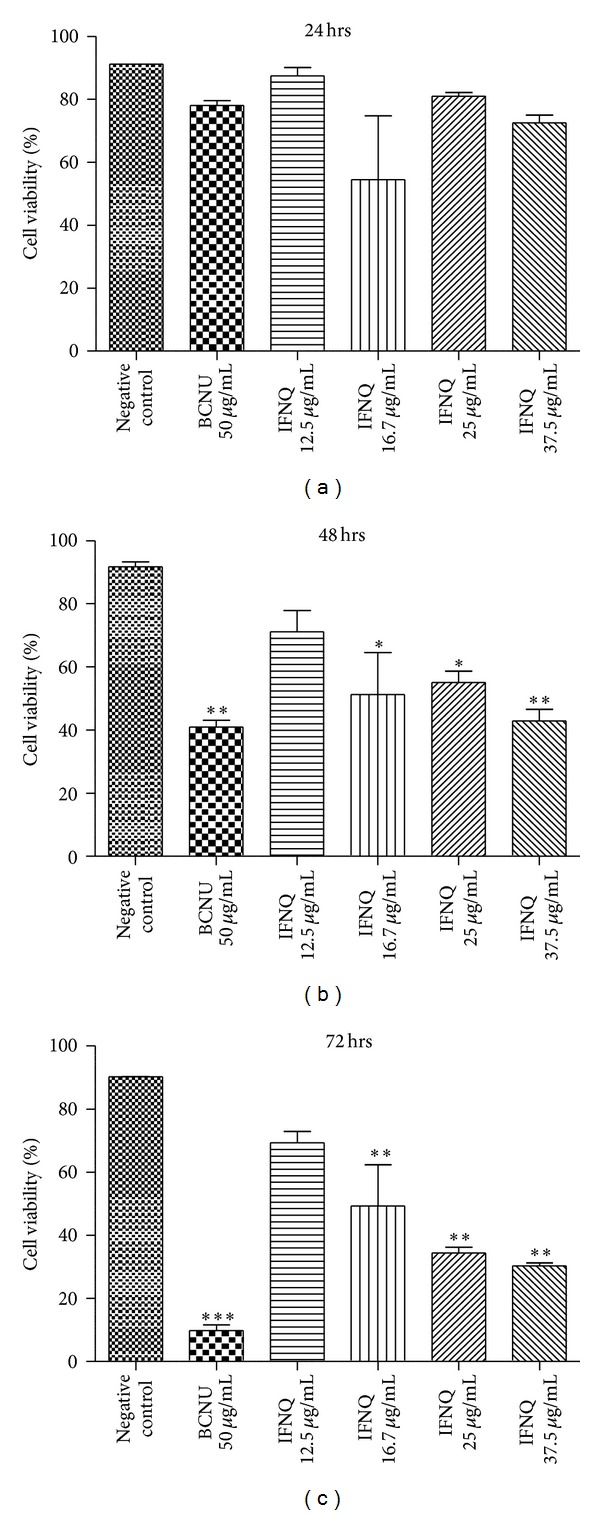
The effects of different concentrations of IFNQ on Jurkat T cells. Different concentrations of IFNQ of 12.5 *μ*g/mL, 16.7 *μ*g/mL, 25 *μ*g/mL and 37.5 *μ*g/mL were used in individual wells and wells containing the negative control and positive controls (containing BCNU). Results are the mean of ± SD for *n* = 2 for duplicate measurements. ***P < 0.0001, **P < 0.001, and *P < 0.05.

The effects of isofuranonaphthoquinone on Jurkat T cells were determined by assessing for cell viability. Initially the cells had been exposed to a highest concentration of isofuranonaphthoquinone of 50 *μ*g/mL which was shown to kill all cells (result not shown). The highest concentration was then reduced to 37.5 *μ*g/mL (Figure 2) as this concentration gave a viability of at least 75% after 24 hours. Percentage cell viability decreased with increasing isofuranonaphthoquinone concentration with time from 24 hrs to 72 hrs compared to the negative control which had a viability of 90% at 72 hrs, while the viability in the positive control decreased to 8.5% at 72 hrs. However, for the isofuranonaphthoquinone, a significant decrease was observed at 25 *μ*g/mL where the viability was 34.4% after 72 hours.

### 3.2. Effect of Combining IFNQ with BCNU

**Figure 3:**
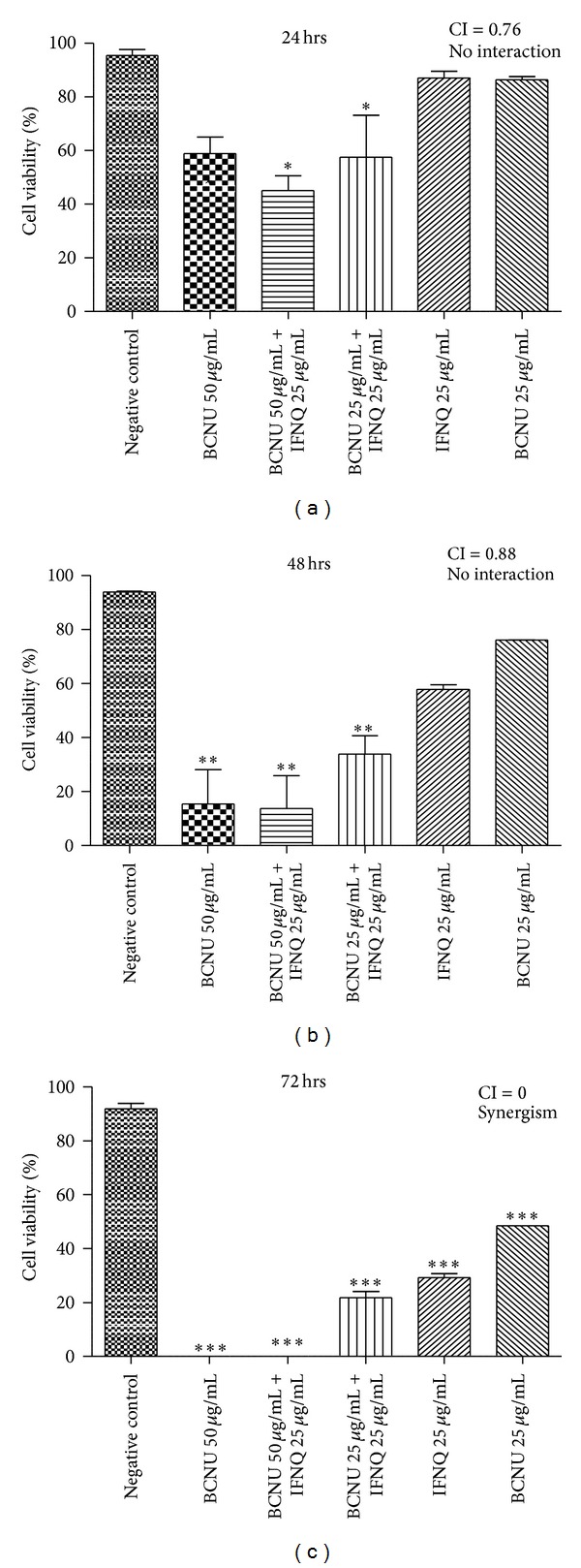
The effects of combining IFNQ and BCNU on Jurkat T cells. IFNQ at 25 was combined with BCNU at 25 or 50 *μ*g/mL. Results are the mean of ± SD for *n* = 2 for duplicate measurements. ***P < 0.0001, **P < 0.001, and *P < 0.05. Key: values <0.5: synergism, >0.5 to 1.0: no interaction, and 1.0 to 4.0: antagonism [4].

When BCNU (25 and 50 *μ*g/mL) was combined with isofuranonaphthoquinone (25 *μ*g/mL) the percentage cell viability decreased more than the effect elicited the individual compounds suggesting a synergistic effect (Figure 3). In this combination, the viability was reduced to 0%. The combination index value showed that there was a synergistic effect (CI = 0) between isofuranonaphthoquinone and BCNU and, hence, accelerated the death of cells with a resultant decline in cell viability. Isofuranonaphthoquinone at a concentration of 25 *μ*g/mL resulted in a cell viability of 29.2% (Figure 3), while in the negative control, the cell viability was 92.1% after 72 hrs.

### 3.3. Effect of Combining the Isofuranonaphthoquinone with Reduced Glutathione

**Figure 4:**
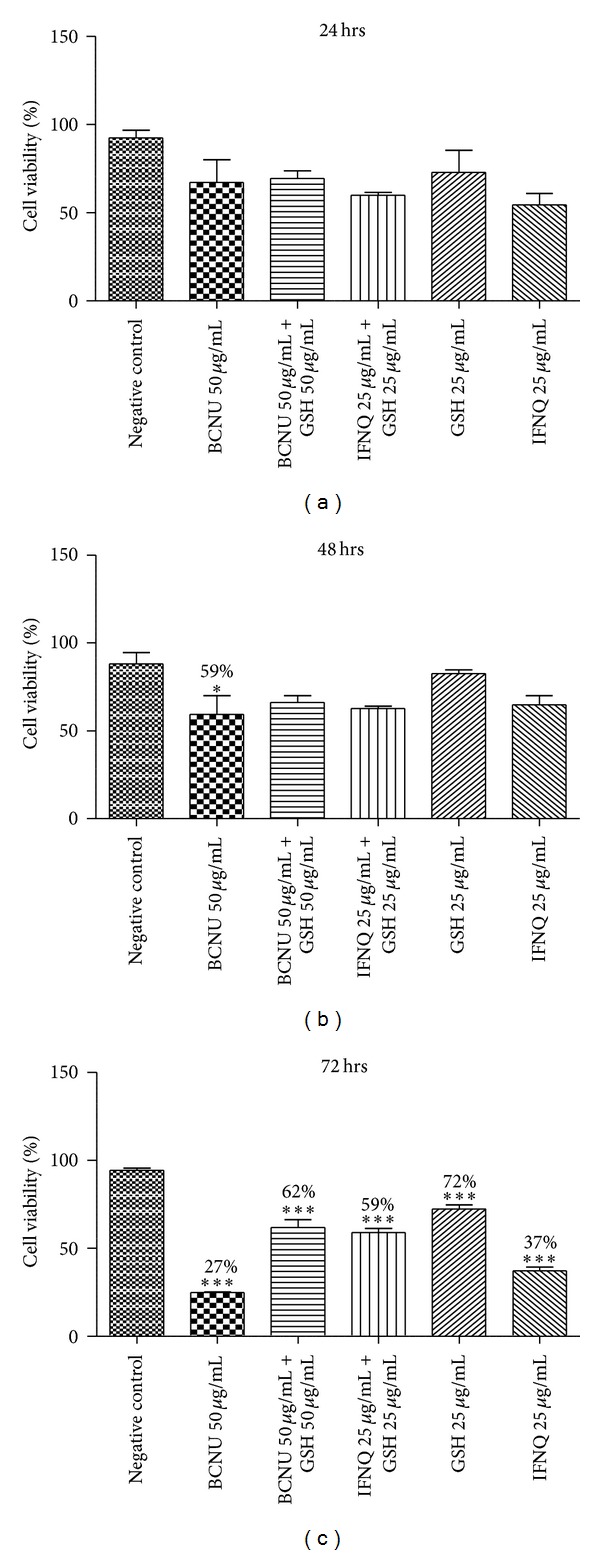
The effects of combining IFNQ or BCNU with reduced glutathione on Jurkat T cells. GSH at equivalent concentrations was combined with BCNU (50 *μ*g/mL) or IFNQ (25 *μ*g/mL). GSH and IFNQ were also incubated singly at 25 *μ*g/mL. Mean percentage values are shown for each concentration. Results are the mean of ± SD for *n* = 2 for duplicate measurements. ***P < 0.0001, **P < 0.001, and *P < 0.05.

When the isofuranonaphthoquinone was combined with GSH, the percentage cell viability observed at 72 hrs decreased to 62%. While cells with GSH alone had a viability of 72% after 72 hrs, isofuranonaphthoquinone-treated cells produced 37% viability. The presence of GSH resulted in an increase in percentage cell viabilities as compared to its absence (Figure 4). It was also noted that cells at 50 *μ*g/mL of BCNU + 50 *μ*g/mL GSH as well as at 25 *μ*g/mL IFNQ + 25 *μ*g/mL GSH had the same percentage viability at both 48 and 72 hours.

### 3.4. Determination of the Reversibility of the Effects of Isofuranonaphthoquinone

**Figure 5:**
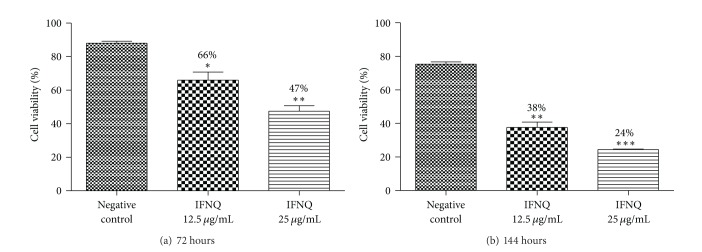
The effects of IFNQ on Jurkat T cells at 72 and 144 hours of incubation. Cells were incubated with IFNQ at 12.5 or 25 *μ*g/mL and counted after 72 hours (a). Then the cells were centrifuged, washed twice with HBSS, and incubated in media free of IFNQ for another 72 hours (b). Results are the mean of ± SD for *n* = 2 for duplicate measurements. *P < 0.05.

Results of the assay to determine whether the effects of isofuranonaphthoquinone on Jurkat T cells are reversible are shown in Figure 5. After 72 hrs, the cell viabilities for negative control (0 *μ*g/mL), 12.5 *μ*g/mL, and 25 *μ*g/mL isofuranonaphthoquinone were 88.1%, 66.0%, and 44.7%, respectively. After the cells were washed with RPMI to remove isofuranonaphthoquinone and incubated again for another 72 hours, the cell viabilities obtained after 144 hrs for 0, 12.5, and 25 *μ*g/mL of IFNQ were 75.4%, 37.6%, and 24.5%, respectively. The cell viabilities for the 12.5 *μ*g/mL and the 25 *μ*g/mL were not comparable to the negative control which had not been treated with IFNQ, thus indicating that the effects of isofuranonaphthoquinone were not reversible.

### 3.5. Effects of Isofuranonaphthoquinone on GST Activity 

**Figure 6:**
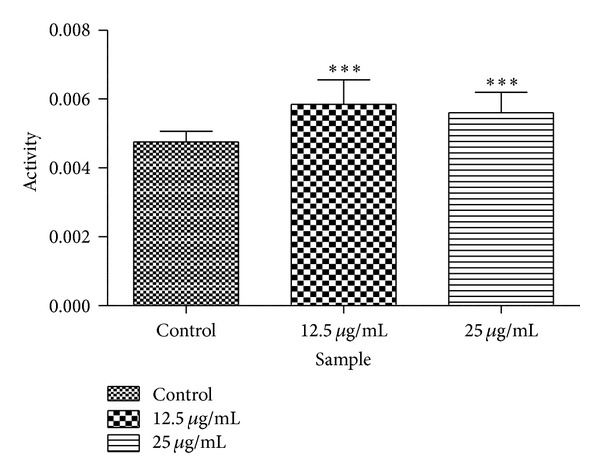
The effect of C. platypetalum on glutathione transferase cytosolic activity in Jurkat T cells. Cells were incubated with different two concentrations of the IFNQ in a 96-well plates. After cells were lysed and cytosolic GST activity was determined using the substrate 1-chloro-2,4-dinitrobenzene. Values represent the mean ± SD for *N* = 24. ***P < 0.0001.

The effects of isofuranonaphthoquinone on GST activity were assessed by measuring the conjugation activity with CDNB in the absence and in the presence of IFNQ. GST activity is normally elevated in cancer cells due to the overexpression of the genes that code for the GSTs. Activities of 0.0047 ± 0.0003, 0.0058 ± 0.0007, and 0.0056 ± 0.0006 units products/mg protein were obtained for 0, 12.5 *μ*g/mL and 25 *μ*g/mL IFNQ, respectively, (Figure 6). These increases in activity were significant (*P* < 0.05) as compared to the control.

### 3.6. Effect of Isofuranonaphthoquinone on Drug Efflux Pumps

**Figure 7:**
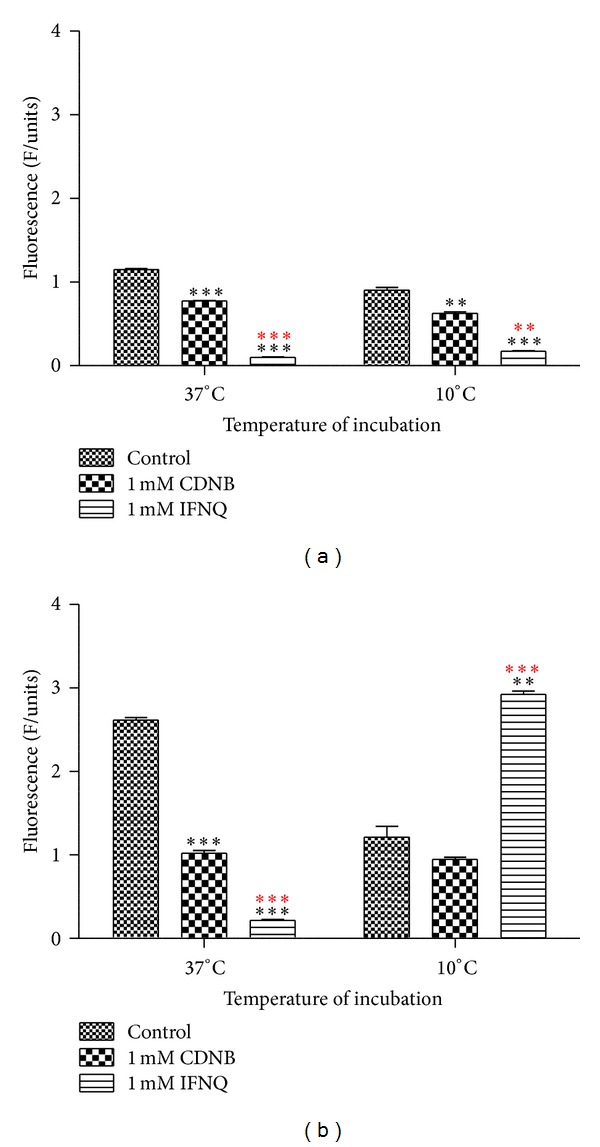
The effects IFNQ on drug efflux of the MCB-GSH conjugate from Jurkat T cells as a function of treatment. Cells were incubated with MCB. CDNB (positive control) or the IFNQ was added. Cells were then spun and the supernatant taken to determine the amount of the MCB-GSH conjugate effluxed (a). Cells were also lysed and the amount of MCB-GSH in the cells was also determined (b). Preincubations were performed at 10°C and 37°C. Values are the mean of ± SD for *n* = 2 for duplicate measurements ***P < 0.0001, **P < 0.001, and *P < 0.05. ***Asterisks in black are differences with the control, whilst those in red are differences between CDNB and IFNQ.

**Figure 8:**
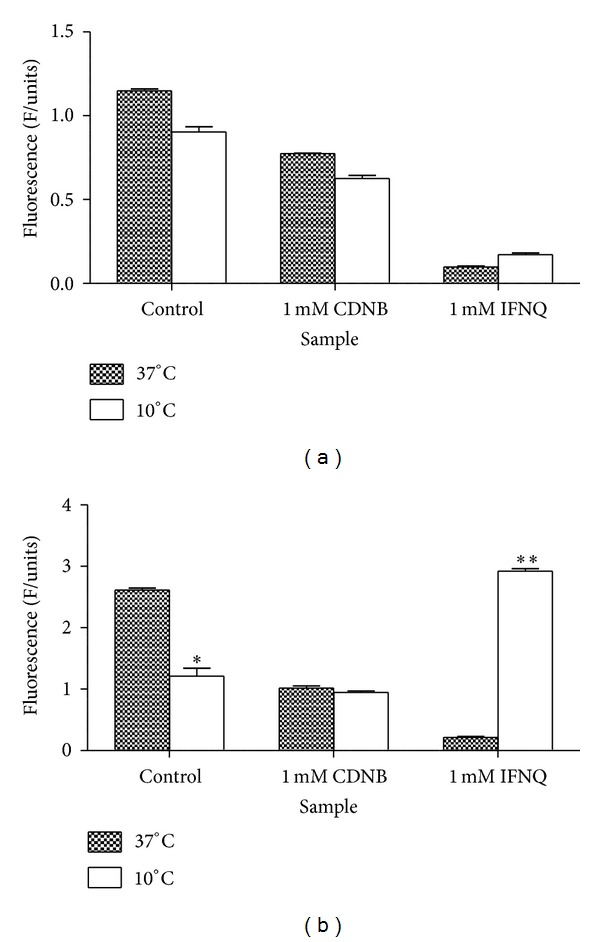
The effects IFNQ on drug efflux of the MCB-GSH conjugate from Jurkat T cells as a function of temperature of incubation. (a) Amount of conjugate effluxed. (b) Amount of conjugate accumulated. Values are the mean of ± SD for *n* = 2 for duplicate measurements **P < 0.001 and *P < 0.05.

The effect of IFNQ on drug efflux pumps was determined using MCB and CDNB as substrates of glutathione transferase. MCB is a fluorescent and was used to probe the activity of the efflux of the pumps. CDNB was used as a positive control at 1 mM to inhibit the activity of the pumps. IFNQ was also used at a concentration of 1 mM. Multidrug resistance associated proteins (MRPs, GSX pumps) are responsible for the efflux of conjugates from cancer cells. MCB-GSH conjugate accumulation outside the cell was determined using the fluorescence of MCB-GSH in supernatants using a Shimadzu spectrofluorometer (Shimadzu UV-1501, Japan) (Figure 7(a)). The cells were then sonicated to break open the cell membrane after which the accumulated GSH-MCB conjugate was measured (Figure 7(b)).  Figure 8(a) shows that temperature had no effect in terms of the amount of MCB-GSH that was effluxed out of the cells. However, when it comes to the amount of MCB-GSH accumulated (Figure 8(b)), temperature had a significant effect. The levels of MCB-GSH were lowered in the control at 10°C but increased in cells exposed to 0.025 mg/mL IFNQ at the same temperature.

## 4. Discussion

Cancer is a major public health burden in both developed and developing countries. The prevalence of cancer has been on the rise with one in four people being expected to develop cancer every year [36]. The search for safe and efficacious agents for use in the medical oncology field is ongoing with the objective being to find new drugs which are efficacious but with fewer side effects. The conventional medicines have been shown to produce greater side effects and, thus, efforts are being put into research. Natural products have played an important role in the development of contemporary cancer chemotherapy [32]. In this study, a leukemic cell line was used to determine the anticancer effects of a natural plant product from *Bulbine frutescens*. Jurkat T cells are an immortalized T lymphocytes cell line used to determine the mechanism of action of anticancer agents [37, 38].

During preliminary studies, the cells were exposed to a concentration of 50 *μ*g/mL isofuranonaphthoquinone and this resulted in death of all the cells (data not shown) and this prompted the use of lower concentrations of 37.5 *μ*g/mL (75% viability) for successive experiments. The Trypan blue assay was used to determine cell viability. In the negative control, the cell viability did not change significantly, 91.2% after 24 hrs and 90.4% after 72 hrs. At concentrations of 50 *μ*g/mL, isofuranonaphthoquinone resulted in 0% cell viability, while BCNU, the positive control at this concentration, resulted in a cell viability of 9.75%. Thus, isofuranonaphthoquinone from *B. frutescens *possessed greater antiproliferative activity than BCNU, at equal concentrations. It is noteworthy that the cell viabilities for the same experiments (Figures 2–5) employing 25 *μ*g/mL after 72 h have arguably notable differences—34.4%, 29.2%, and 37% versus 44.7%, respectively. Importantly, it was observed that trends were the same in each set of experiments. A plant called *Ventilago madraspatana* has been shown to contain phytochemicals that include isofuranonaphthoquinones, ventilone-c, ventiloquinones E, G, and J, Eleuthrin, enantiopure 1, and 3 dimethyl pyranonappthoquinones [39]. In a preliminary study carried out by Chittethu et al., [40], they showed that *Ventilago madraspatana* had moderate cytotoxicity towards the MCF-7 cell line. The authors indicated that this could have been due to the presence of isofuranonaphthoquinones, flavonoids, and alkaloids. In another study, 1-naphthol, 1,2-naphthoquinone, and 1,4-naphthoquinones were shown to be selectively toxic to short-term organ culture of human colonic tumor tissue [41]. Naphthoquinones are, therefore, a potential source of anticancer drugs.

**Figure 9:**
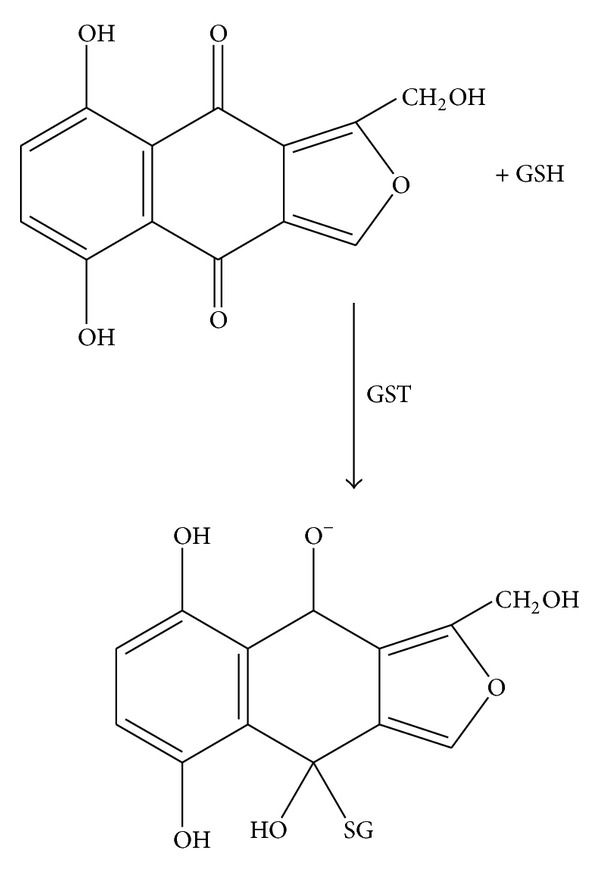
Proposed putative reaction of the isofuranonaphthoquinone and glutathione.

Quinones have been shown to possess anticancer activity and many clinically useful antitumor agents including adriamycin, daunorubicin, actinomycin, and mitomycin C contain a quinone or quinone-like moiety in their structure [42, Figure 1]. The antitumor activity of naturally occurring quinones is exhibited predominantly by three main groups, namely, benzoquinone, naphthoquinone, and anthraquinone [43]. Cancer cells are particularly vulnerable to treatments impairing redox homeostasis [44]. Isofuranonaphthoquinone at a concentration of 25 *μ*g/mL was combined with 50 *μ*g/mL glutathione to determine if impairment of redox homeostasis was a possible mechanism of action for this compound. GSH is a scavenger of free radicals such as reactive oxygen species (ROS) and reactive nitrogen species (RNS) and in its presence, there is no accumulation of the oxidants. Since glutathione is a powerful intracellular antioxidant [45], it was expected that if the IFNQ was working via a redox mechanism then its effects would be antagonized by glutathione resulting in increased cell viability. A percentage viability of 37% for 25 *μ*g/mL for IFNQ versus 72% for 25 *μ*g/mL GSH and 59% for 25 *μ*g/mL GSH + 25 *μ*g/mL IFNQ (Figure 4). Clearly the GSH was protecting cells against the effects of IFNQ. Similar results were obtained for BCNU. It has been shown that cells become less sensitive to anticancer agents in the presence of glutathione [46]. Isofuranonaphthoquinone was possibly exerting its anticancer activity on Jurkat cells by generating ROS which resulted in cell death. Since there was an increase in cell viability in the presence of GSH, produced ROS were probably quenched by the GSH. In cancer therapy, alkylating agents and radioactive isotopes are known to kill cancer cells by the mechanism of oxidative stress [47]. Alternatively, the GSH could be reacting directly with the IFNQ (Figure 9) as this is a standard reaction of quinones and reduced glutathione [33]. Normal cells possess antioxidant systems to cope with ROS and prevent their damaging effects. They are able to maintain redox homeostasis with a low level of basal ROS because they tightly control the balance between ROS generation and elimination [48]. On the contrary, cancer cells exhibit an abnormal redox status associated with increased basal levels of ROS and alterations of the antioxidant systems [49]. As a consequence, normal cells can tolerate a certain level of exogenous ROS whereas cancer cells cannot. This is the basis of using compounds that generate ROS as there will be selective toxicity [50]. The intracellular ROS-scavenging system includes superoxide dismutases (SOD), glutathione peroxidase (GPx), peroxiredoxins (PRDXs), glutaredoxins, thioredoxins (TRXs), glutathione transferases, and catalases [51]. While controlled ROS levels exert a prosurvival effect in cancer cells by acting as essential intracellular second messengers for cytokines and growth factors, high ROS levels can cause cellular damage, and even cell death [49]. Although cancer cells develop adaptive mechanisms to minimize the effects of oxidative damage excessive ROS levels can disrupt redox homeostasis and, hence, affect the survival of the cancer cells by irreversibly damaging cellular macromolecules such as DNA, carbohydrates, protein, and lipids [50].

In clinical practice, cancer drugs are used in combination of two or more drugs for the treatment of cancer. In this study, the effect of combining isofuranonaphthoquinone with a standard anticancer drug BCNU was investigated. Cell viability decreased to 0% after BCNU 50 *μ*g/mL was combined with isofuranonaphthoquinone 25 *μ*g/mL for 72 hrs, while BCNU alone resulted in a cell viability of 9.75%. The study showed that isofuranonaphthoquinone and BCNU have synergistic effects at these concentrations after 72 hrs with a combination index of 0. These results show that the two may be used together for an effective killing of cancer cells. Exinger et al. [52] mentioned the use of 5-Fluorouracil in combination with leucovorin to positively modulate thymidylate synthase inhibition. In another study, low-dose cisplatin and 5-Fluorouracil were shown to be effective in combination than when used individually against chemoresistant gastrointestinal cancer [53]. In a study to determine the effects of Zimbabwean medicinal plants on leukemia cell lines, Mukanganyama et al. [4] showed that the methanolic extract of *Parinari curatellifolia* had synergistic effects with doxorubicin at equal concentrations of 10 *μ*g/mL (combination index = 0). The synergistic mechanisms observed when cancer drugs are used in combination enhance therapeutic outcomes. Thus IFNQ can, therefore, be used in combination with other drugs to produce effective suppression of cancer cells.

GSTs are involved in the detoxification of several chemotherapeutics and are crucial in regulating the susceptibility to cancer. Some studies have shown that GSTs are expressed at higher levels in drug-resistant cancer cell lines compared with normal tissues [54, 55]. The effect of isofuranonaphthoquinone on GST activity was determined and our results show that there was an increase in GST activity at a concentration of isofuranonaphthoquinone of 12.5 *μ*g/mL as compared to the control (*P* < 0.05). The increase in GST activity could have been due to an induction of the GSTs by the compound. The human body has evolved inducible metabolizing enzymes and efflux transporters [56]. These are responsible for facilitating the metabolism and elimination of potentially harmful drugs and xenobiotics. Morgan et al. [57] reported that modulation by inhibition of GST has been attempted as a means to improve response to cancer drugs. Chemomodulation by the administration of nontoxic agents can be an effective method to improve therapeutic outcomes in cancer patients by inhibiting GST activity where the cancer drugs used are metabolized by GSTs [58]; this might be accomplished by inhibiting glutathione synthesis [59]. Ethacrynic acid, a diuretic, has been shown to counter GSTP1-1 activity and, hence, it can be used in chemotherapy to increase the effectiveness of alkylating cancer drugs [60]. van Zanden et al. [61] showed that quinone-type oxidation products of quercetin are potent inhibitors of GSTP1-1 activity; quercetin is a flavonoid. The quercetin quinone was shown to play a role in the inactivation of GSTP1-1 through covalent binding to the cysteine residue. However, the inhibition of GSTP1-1 was not seen when coincubated with ascorbic acid or glutathione as these probably prevented the formation of oxidation products of quercetin. Mukanganyama et al. [33] showed that isofuranonaphthoquinone was a potent inhibitor of GSTs *in vitro* and, therefore, the results of this study on the effects of this compound in cancer cell which usually overexpress GSTs become of paramount importance. The effect of isofuranonaphthoquinone as an inhibitor of GSTs could make it desirable for use in combination with other standard anticancer drugs which are susceptible to metabolism by GSTs.

**Figure 10:**
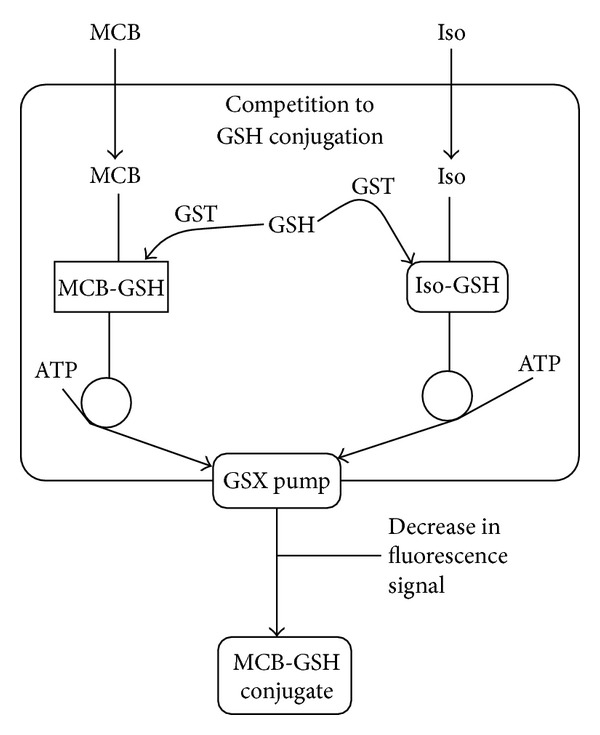
Schematic diagram of GSH-based detoxification pathway. MCB is used as a model substrate for conjugation to GSH by GST in the cytosol and there is subsequent sequestration by a GSX pump. MCB is not fluorescent until conjugated to GSH giving the MCB-GSH conjugate. If another xenobiotic substrate for conjugation is present such as isofuranonaphthoquinone (Iso), competition will occur (Iso-GSH), resulting in the decrease in fluorescence signal from MCB-GSH conjugate formation.

**Figure 11:**
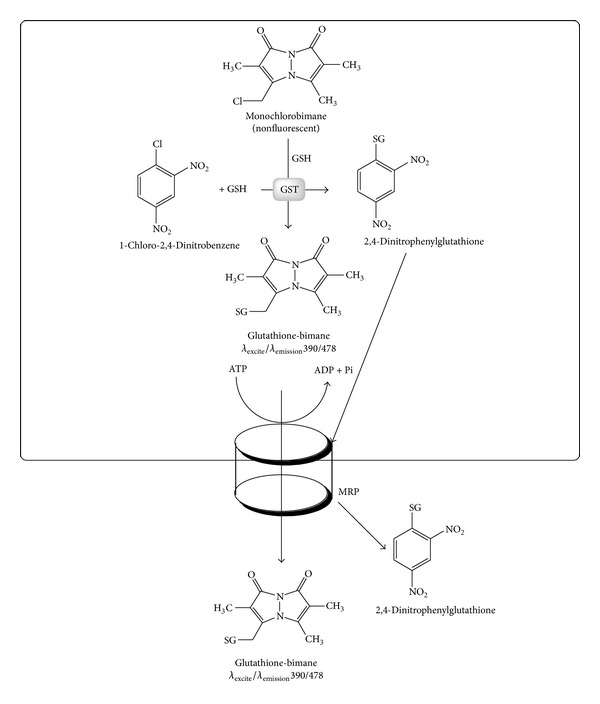
The reactions of MCB, CDNB, and glutathione in a reaction catalysed by glutathione transferases. The MCB-GSH and the CDNB-GSH conjugates can both be effluxed by MRP proteins. Competition between MCB and CDNB, therefore, is both at GST-mediated catalysis as well as MRP-mediated efflux.

A wide range of hydrophobic substances are eliminated from the cytosol to the extracellular space after their conjugation with GSH [62]. This transport is mediated by a novel class of organic anion transporters belonging to the family of ATP-binding cassette (ABC) carriers, the GS-X pumps, or multidrug resistance associated proteins. Tumor cells overcome the challenge of anticancer drugs and biologically active hydrophobic substances of an endogenous nature by exporting them via different unidirectional efflux systems [63]. Multidrug resistance often occurs during cancer treatment when a tumor that was originally sensitive to treatment becomes resistant [64]. This is a major obstacle in achieving effective treatment outcomes and often leads to poor prognosis and death. Glutathione S-conjugate export (GSX) pumps are implicated in the development of drug resistance and the efflux of xenobiotics as conjugates of glutathione [65]. Isofuranonaphthoquinone was shown to inhibit drug efflux pumps at a concentration of 25 *μ*g/mL significantly (*P* < 0.05) when incubated at 37°C using monochlorobimane (MCB) as a substrate. In the presence of 1-chloro-2,4-dinitrobenzene (CDNB), less MCB-glutathione conjugate was effluxed (Figure 7). Isofuranonaphthoquinone could possibly be an inhibitor of the efflux pumps, hence leading to accumulation of MCB inside the cell. It is possible that CDNB conjugated to glutathione and competed for efflux by the same pumps that effluxed MCB-glutathione conjugate and this could have reduced drug efflux (Figure 10). However, the drug accumulated did not complement the efflux of MCB when considering IFNQ (Figure 7(b)). There was less MCB-GSH accumulated in cells which could also have been due to inhibition of the GSTs by isofuranonaphthoquinone. In addition it was also shown that IFNQ had a more significant effect on drug efflux or accumulation compared to CDNB as there were significant differences in the levels of MCB-GSH after exposure to the two compounds. The accumulation of MCB-GSH in the cells was affected by temperature (Figure 8). In the presence of IFNQ, the accumulation increased at 10°C. At 37°C, the formation of MCB-GSH conjugate is an enzyme catalyzed reaction catalyzed by GSTs and, hence, inhibition of the latter could possibly result in a reduction of the formation of the conjugate (Figure 11) or the conjugate was being effectively effluxed out of the cell. At 10°C, the transport of the conjugate could have been affected by the temperature as this is an enzyme catalysed reaction. The isofuranonaphthoquinone is, therefore, a potent inhibitor of drug efflux pumps.

In conclusion, our study shows that the isofuranonaphthoquinone isolated from *Bulbine frutescens* possesses antiproliferative effects on Jurkat T cells. The effect was comparable to the anticancer agent BCNU. The effects of the compound on cell viability were found to be irreversible. The study also showed that isofuranonaphthoquinone could be exerting its anticancer activity by generating reactive oxygen species resulting in cell death. A combination of isofuranonaphthoquinone with a standard anticancer drug BCNU was shown to produce greater toxicity effects on the Jurkat T cells than the individual compounds. The isofuranonaphthoquinone was also shown to inhibit drug efflux pumps which have been implicated in drug resistance in cancer cells. Therefore, this compound has potential as a lead candidate in the development of anticancer agents or as an adjunct compound in combination treatment regimes.

## Abbreviations

DMSO: Dimethyl sulfoxide
BCNU: 1,3-Bis(2-chloroethyl)-1-nitrosourea
IFNQ: Isofuranonaphthoquinone
GST: Glutathione transferases
MCB: Monochlorobimane
GSH: Reduced glutathione
HBSS: Hank's buffered salt solution
RPMI: Roswell Park Memorial Institute
CDNB: 1-Chloro 2,4-dinitrobenzene.
